# Laser Engineered Net Shaping of Nickel-Based Superalloy Inconel 718 Powders onto AISI 4140 Alloy Steel Substrates: Interface Bond and Fracture Failure Mechanism

**DOI:** 10.3390/ma10040341

**Published:** 2017-03-25

**Authors:** Hoyeol Kim, Weilong Cong, Hong-Chao Zhang, Zhichao Liu

**Affiliations:** 1 Department of Industrial, Manufacturing and Systems Engineering, Texas Tech University, Lubbock, TX 79409, USA; weilong.cong@ttu.edu (W.C.); hong-chao.zhang@ttu.edu (H.-C.Z.); zhichao.liu@ttu.edu (Z.L.); 2 School of Mechanical Engineering, Dalian University of Technology, Dalian 116023, China

**Keywords:** laser additive manufacturing, tensile test, fractography, metallurgical defects, elemental segregation, Laves phase, oxide formation

## Abstract

As a prospective candidate material for surface coating and repair applications, nickel-based superalloy Inconel 718 (IN718) was deposited on American Iron and Steel Institute (AISI) 4140 alloy steel substrate by laser engineered net shaping (LENS) to investigate the compatibility between two dissimilar materials with a focus on interface bonding and fracture behavior of the hybrid specimens. The results show that the interface between the two dissimilar materials exhibits good metallurgical bonding. Through the tensile test, all the fractures occurred in the as-deposited IN718 section rather than the interface or the substrate, implying that the as-deposited interlayer bond strength is weaker than the interfacial bond strength. From the fractography using scanning electron microscopy (SEM) and energy disperse X-ray spectrometry (EDS), three major factors affecting the tensile fracture failure of the as-deposited part are (i) metallurgical defects such as incompletely melted powder particles, lack-of-fusion porosity, and micropores; (ii) elemental segregation and Laves phase, and (iii) oxide formation. The fracture failure mechanism is a combination of all these factors which are detrimental to the mechanical properties and structural integrity by causing premature fracture failure of the as-deposited IN718.

## 1. Introduction

American Iron and Steel Institute (AISI) 4140 alloy steel has been widely used for the manufacturing of gear components due to its good performance of strength, toughness, and wear resistance [1]. Due to the harsh working conditions of the components made of this alloy steel, it is necessary to extend their service life and refurbish the worn or damaged parts [2]. However, when they are exposed to harsh working environments such as high temperature, high speed sliding contact, and frictional heat generation, their surfaces undergo severe damage such as micropitting of gear teeth flanks and tooth breakage in the tooth root due to repeated cyclic sliding loading under long-term service, which is detrimental to the service life of the critical gear components and leads to the complete failure of the gear [3,4].

Traditionally, once this failure happens, the damaged gear is discarded resulting in serious resource waste and the loss of high added value in gear manufacturing. Considering the high costs and long lead time for the replacement of the failed components, more effective repair technology is needed [5]. There also exists a high demand in industry to improve the performance and durability of all the components [6], and it is imperative to repair and restore these worn-out or damaged components so as to extend the service life and thereby reduce the overall cost [7].

Recently, surface treatment processes such as carburizing [8], nitriding [9], and boriding [10] have been commonly used to enhance surface integrity and properties such as surface hardness, wear, corrosion, and oxidation resistance [11]. However, these processes are rather difficult to control and are very time consuming.

Laser engineered net shaping (LENS) is a laser additive manufacturing process that uses high power laser as a heat source to create a melt pool on the surface of a solid substrate and melt metal alloy powders through powder feeding nozzles. Not only can it fabricate a complex, functional, and structural part, but also can be used for surface treatment such as coating, hardfacing, as well as repairing worn and damaged parts. Additionally, it has shown excellent metallurgical bonding to the substrate with a minimum heat affected zone (HAZ) compared to other surface coating processes such as high-velocity oxy fuel spraying (HVOF), plasma spraying (PS), or tungsten inert gas (TIG) welding [12,13,14]. Thus, these coatings/repairs by LENS not only enhance the lifetime of the components subjected to severe working conditions but also provide reduced downtime and costs.

Nickel-based superalloy Inconel 718 (IN718), a precipitation hardening alloy, is one of the widely used high-performance materials in the aerospace industry due to good mechanical performance at elevated temperature including wear, corrosion, and oxidation resistance. Thus, it can also be used as a prospective candidate material for surface coating and repair applications when the LENS process is utilized.

LENS of IN718 has been studied by researchers to investigate its microstructure and mechanical properties [15,16,17]. However, to date there has been no available research on LENS-deposited IN718 powders on AISI 4140 alloy steel substrates in the literature. No published literature was available on the interface bond and mechanical properties of a hybrid part which is made up of half-deposited material and half-substrate material. Joining two dissimilar materials with a good interfacial bond is critical to ensure the overall performance of the whole part, since low bond strength may lead to failure resulting from the coating peeling off, cracking, or corrosion along the interface [5]. The coated or repaired material will be functional only when the interface between the coating and the substrate is durable and strong [18].

Therefore, the aim of this study is to investigate the compatibility of LENS-deposited IN718 with AISI 4140 alloy steel substrate with a focus on interface bond performance and fracture behavior of the hybrid fabricated test specimens. To do so, a tensile test is carried out to examine the interfacial bond and fracture behavior of the hybrid specimens. To elucidate the fracture failure mechanism, the cross-sectional interface and fracture surfaces are characterized using scanning electron microscopy (SEM) and energy disperse X-ray spectrometry (EDS).

## 2. Materials and Methods

AISI 4140 low alloy steel was used as the substrate material in the form of a round bar with dimensions of 9 mm diameter and 30 mm length. The chemical composition (wt %) of the substrate was C 0.42, Si 0.25, Mn 0.80, S 0.02, Cr 1.05, Mo 0.20, and Fe 97.26. The surface was first ground with abrasive papers to remove oxidation layers and then cleaned with acetone prior to deposition. Commercially available gas-atomized (GA) IN718 powder with a particle size ranging from 44 to 125 µm was utilized in the as-received condition. Chemical composition (wt %) of the powders was Al 0.3, Ti 0.6, Mo 2.8, Nb 4.7, Cr 19.0, Fe 19.0, and Ni 53.6.

**Figure 1:**
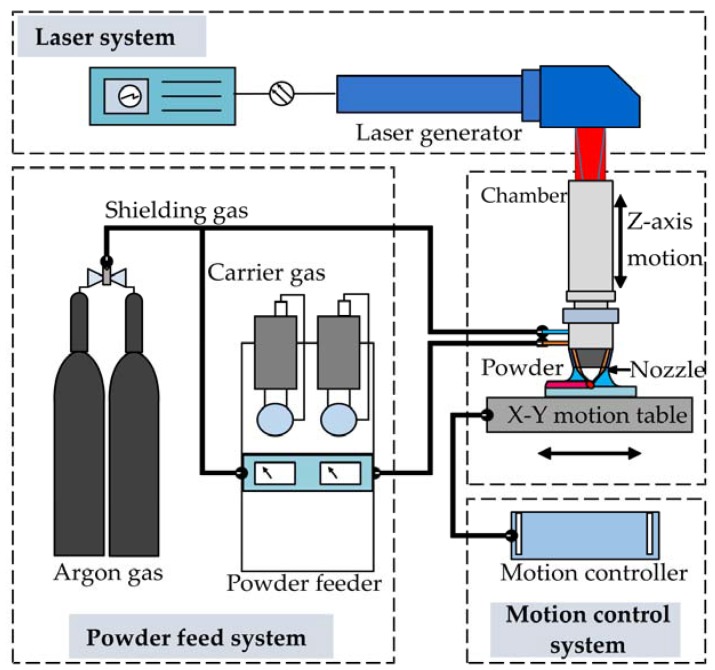
Schematic illustration of the LENS system used in this study.

The experiment was carried out using an Optomec LENS 450 system, equipped with a maximum output 400 W IPG fiber laser, a pneumatic powder feed system, and a computer-controlled motion system. The powder feed system is controlled by a computer and motorized with revolutions-per-minute (rpm) adjustment to meter the amount of powder feeding flow. The powder is conveyed by argon as a carrier gas from powder feeders and deposited on a substrate through coaxial nozzles on a four-jet deposition head. A high power laser is used to melt the metal powder at the focus of the laser beam, and the motion table is moved to fabricate an object layer by layer. The complete LENS system used in this study is illustrated in Figure 1.

**Figure 2:**
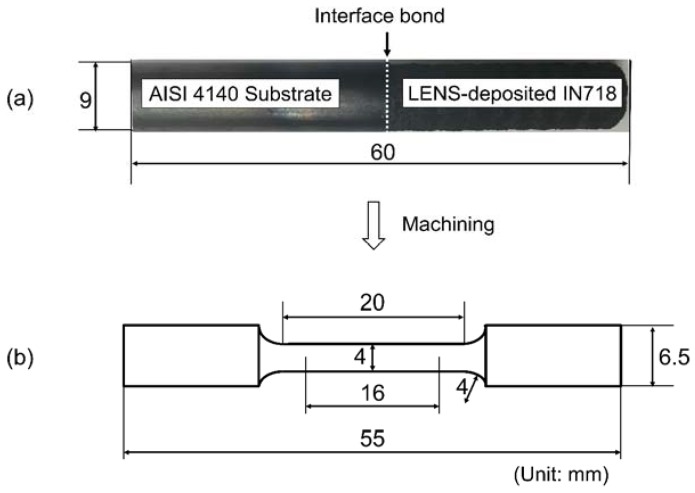
(**a**) Cylindrical pillar after LENS deposition and (**b**) Dimensions of the machined hybrid tensile test specimen.

Based on a series of preliminary experiments, the selected process parameter values were as follows: laser power 310 W, scanning speed 8.47 mm/s, powder feed rate 4 rpm, layer thickness 0.51 mm, carrier gas flow rate 6 L/min, hatch spacing 0.76 mm, and hatch angle 60°. A cylindrical pillar of IN718 powder with the same dimensions as the substrate was deposited directly on the top surface of the substrate (Figure 2a), and then the fabricated part was machined to the final test specimen dimensions according to American Society for Testing and Materials (ASTM) E8-09 Small-Size Specimens Proportional to Standard (Specimen 4), as illustrated in Figure 2b. A total of five specimens were fabricated with the same process conditions and dimensions, and room temperature tensile tests were conducted with 4 mm diameter and 16 mm gauge length round specimens using an Instron MTS universal tester.

A metallographic sample was prepared from the cross-sectional interface part between the substrate and the as-deposited IN718 based on a standard procedure. The cross-sectional sample was ground with SiC grit paper, polished with 0.05 µm Al_2_O_3_ finish, and etched by Kalling’s reagent (5 g CuCl_2_ in 100 mL HCI and 100 mL ethanol). Metallurgical bonding at the interface, morphology, and defects were examined by SEM with Hitachi S-4300 and Zeiss Crossbeam 540. To investigate the fracture failure mechanism, fractography analysis was carried out to analyze the fracture surfaces of the hybrid fabricated part after the tensile test using the same SEM equipment. Quantitative chemical composition analysis using EDS was also implemented to identify elemental segregation and phase formation.

The average microhardness profile was measured by a Vickers microhardness (HV) tester (Phase II) on the cross-sectional part including the LENS-deposited IN718, interface, and the substrate. A standard test load of 1 kgf was applied for a dwell time of 15 s at an indent interval of 0.25 mm, with three replications on the same hardness measurement lines.

## 3. Results and Discussion

### 3.1. Metallurgical Observation at a Cross Section of the Interface

**Figure 3:**
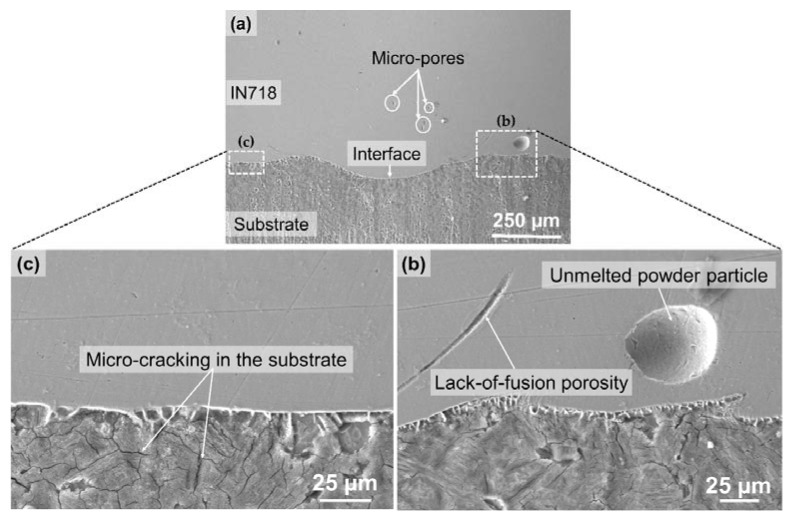
SEM images of (**a**) a cross section of the interface between the AISI 4140 substrate and as-deposited IN718; (**b**) high magnification of the dotted area from (**a**) indicating unmelted powder particle inclusion and lack-of-fusion porosity; and (**c**) high magnification of the dotted area from (**a**) showing micro-cracking of the substrate near the interface due to the rapid thermal gradient.

Figure 3a shows SEM images of a cross-sectional morphology of the interface between the AISI 4140 substrate and the as-deposited IN718 obtained from the hybrid tensile specimen. The interface exhibited smooth metallurgical bonding between the two dissimilar materials with minimal dilution, which is one of the advantages of the LENS process compared to other surface coating processes such as high-velocity oxy fuel spraying (HVOF), plasma spraying (PS), or tungsten inert gas (TIG) welding [12,13,14].

Metallurgical defects such as unmelted powder particle inclusion, micro pores, and lack-of-fusion porosity were observed in the laser-deposited IN718 part near the interface between the laser-deposited layers (Figure 3a,b), which are prevalent in laser-based additive manufacturing processes and have been widely reported in the literature [19,20,21]. These defects could reduce the density of the as-deposited part, resulting in lower tensile properties compared to the corresponding wrought material [22]. During the laser deposition process, the substrate near the interface and HAZ underwent micro-cracking along the grain boundary due to thermal stress caused by the steep thermal gradient between the high temperature melt pool and the chilled substrate, as well as the repeated heat cycles from the laser-deposited layers (Figure 3c).

**Figure 4:**
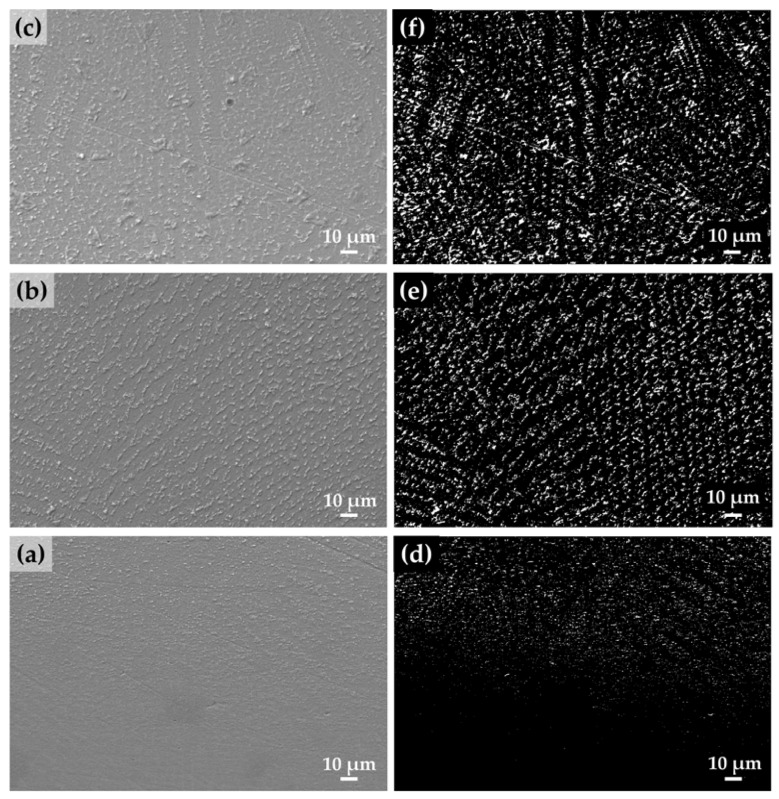
SEM images showing the morphology and concentration of the precipitation of as-deposited IN718 parts in different regions (**a**) bottom; (**b**) middle; and (**c**) upper area, and binary images showing the matrix as black background and precipitation as white particles in (**d**) bottom; (**e**) middle; and (**f**) upper area, corresponding to **a**–**c**, respectively.

Figure 4a–c shows the morphology and concentration of precipitation in different areas of the as-deposited IN718 part for the first eight layers. The small white particles were precipitated along the interdendritic regions, and dendritic arm spacing became larger as the number of deposited layers increased. According to the previous studies [23,24,25,26,27,28], similar precipitation morphology was observed and identified as Laves phase which was segregated from the austenite γ matrix during the rapid solidification of IN718.

The concentration of this white precipitated Laves phase was quantitatively measured in different areas in order to confirm the precipitation changes at the corresponding cross section of the as-deposited IN718 specimen. The gray scale SEM images from Figure 4a–c were binarized as black and white images in which the black background represents the austenite γ matrix and the white particles indicate precipitation (Figure 4d–f). The white area fraction in the bottom area is about 2.5%, but increased to 9.6% in the middle area and 11.6% in the upper area as the height of deposition increased from the substrate. Due to the large thermal gradient near the interface, the precipitation and dendrite growth in the bottom area were suppressed compared to the middle and upper areas. Thus, the precipitation concentration in the upper region was higher than that in the lower region.

According to solidification theory [28,29], the morphology of the dendrite is determined by the ratio of the temperature gradient (G) to the growth rate (R), which is G/R. A columnar dendritic structure is preferred at a lower G/R ratio while equiaxed cellular morphology is preferred at a higher G/R. In the as-deposited part, the upper area has a lower temperature gradient (G) compared with the bottom area. Therefore, the upper and middle area show a columnar-dendritic structure (Figure 4e,f), and the bottom area shows finer cellular morphology (Figure 4d).

During the LENS process, the substrate played a role as a heat sink for the first few layers, which led to the finer cellular morphology. However, the temperature of the as-deposited part becomes higher and the cooling rate becomes lower as the number of deposited layers increases [30]. Thus, the finer cellular precipitated zone disappeared as the number of deposited layers increased since the melt pool temperature increased with the decrease in the heat sink effect of the substrate due to reduced heat conduction to the substrate [31]. This causes the precipitated morphology to become coarser and columnar as the deposited layers become higher due to the increased melt pool temperature, continuous heat flux from the succeeding layers, and heat accumulation in the built part.

It is implied that larger dendrite morphology brings about lower tensile strength according to the Hall-Petch relationship and the Kurz and Fischer relationship [32]. Due to the repeated heat cycling during the process, the as-deposited IN718 part grows coarser and columnar dendritic, which may lead to lower ultimate tensile strength (UTS) and elongation compared to wrought IN718 [22]. As a result, the tensile fracture occurred in the as-deposited IN718 part from the hybrid tensile specimen, indicating inferior tensile strength. The detailed results will be explained in the following section.

### 3.2. Interfacial Bonding and Fracture Behavior

One of the objectives in this study is to investigate the interface bond between two dissimilar materials (i.e., the LENS-deposited IN718 and the AISI 4140 substrate). The interface bond is of primary importance to ensure the compatibility of the IN718 superalloy coating with different substrate materials, as well as structural integrity of the whole hybrid part [5]. Another concern for this hybrid part is that its mechanical properties are expected to be at least equal or comparable to those of the substrate materials [33]. Thus, the tensile test was carried out to examine the interfacial bond and fracture behavior of the hybrid specimens.

**Figure 5:**
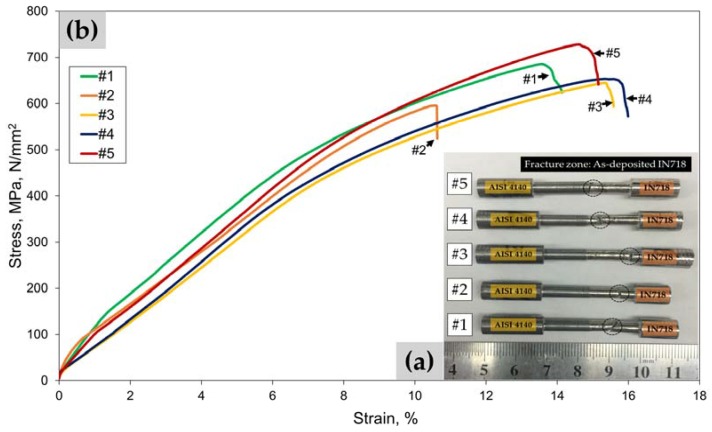
(**a**) Fractured hybrid tensile specimens showing fractures that occurred in as-deposited IN718 marked by dashed circles and (**b**) the corresponding tensile stress-strain curves for each specimen.

All fractured hybrid tensile specimens obtained after the tensile test at room temperature are illustrated with the corresponding tensile stress-strain curves in Figure 5. The left side of the specimen is made of AISI 4140 alloy steel substrate, and the right side of the specimen is made of as-deposited IN718 (Figure 5a). All the specimens failed within the as-deposited IN718 region. No fracture failure was detected on the substrate or the interface, indicating that the as-deposited part of the hybrid specimen is weaker than the AISI 4140 substrate and the interface. Therefore, it can be concluded that the interface bond strength between the AISI 4140 substrate and the as-deposited IN718 is higher than the bond strength between the successive layers of the as-deposited IN718.

The tensile properties of the test specimens were compared with the AISI 4140 substrate and wrought IN718 in Table 1, including ultimate tensile strength (UTS), 0.2% yield stress (YS), and plastic elongation. Since the tensile fractures occurred in the as-deposited IN718, it can be said that the tensile properties of the specimens are representative of the as-deposited IN718. The average UTS (662 MPa) of the hybrid specimens was lower than that of the AISI 4140 substrate (720 MPa).

Furthermore, the average UTS value (662 MPa) obtained from all the test specimens was remarkably lower than the wrought IN718 (1275 MPa). However, the average ductility value (14%) of the test specimens was higher than that of the substrate (4%) and wrought IN718 (12%). When compared to the substrate and wrought IN718, the tensile specimens fractured in the as-deposited IN718 part produced significantly lower UTS and YS, while demonstrating relatively higher ductility. This is because of the lack of strengthening phases (γ″ and γ′) in the as-deposited IN718 caused by the rapid solidification during the LENS process [17,36]. Instead, a significant amount of Nb was segregated from the γ matrix, which depleted it of the Nb necessary to form the strengthening phases. It is reported that heat treatment could improve the UTS and YS of as-deposited IN718, showing even higher values than those of the wrought material by facilitating precipitation of strengthening phases while dissolving segregated Nb and detrimental phases such as Laves and metal-carbide (MC) [17,37]. Therefore, further improvement of the mechanical properties of the as-deposited part would be required to be used for industrial applications.

The prominent necking behavior was observed in the fractured IN718 parts from the hybrid specimens as shown in Figure 5a. Furthermore, the cross-sectional morphology of the fractured specimens varied, such as cone-and-cup or zigzag shapes, indicating a ductile fracture mode. However, no necking was observed from specimen #2 showing the lowest UTS and elongation values compared to the other four specimens (Figure 5b). The fracture surface of specimen #2 was examined and compared with that of specimen #4 showing higher UTS and elongation to investigate the two different fracture behaviors and possible failure mechanisms in detail, as follows.

### 3.3. Fracture Surface and Failure Mechanism

Through the tensile test, all tensile fractures occurred in the laser-deposited IN718 section of the hybrid specimens rather than the interface or the substrate. It is implied that the laser-deposited interlayer bond strength is weaker than interfacial bond strength since tensile fracture normally occurs at the weakest site of the specimen. In order to investigate factors affecting tensile fracture failure in as-deposited IN718, the tensile fracture surfaces were examined using SEM and EDS.

#### 3.3.1. Fracture Morphology and Defects

**Figure 6:**
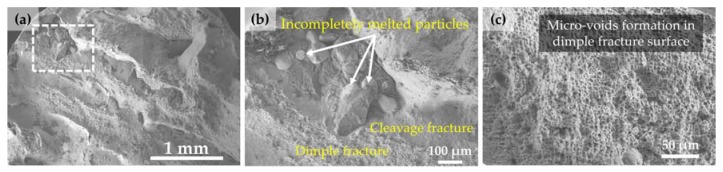
SEM images showing (**a**) overall view of the fracture surface of specimen #2; (**b**) high magnification of a dashed box from (**a**) showing incompletely melted particles and a mixture of ductile and brittle fracture modes; and (**c**) high magnification of the dimple fracture area from (**b**) showing micro-voids formation.

Figure 6a shows the overall fracture surface morphology of specimen #2 that has the lowest UTS and elongation values among the test specimens (Refer to Figure 5b), which appeared to be a mixture of ductile and brittle fracture modes (Figure 6b). The dimple fracture surface with micro-voids formation indicates a ductile fracture (Figure 6c), while the cleavage-type deformation represents a brittle fracture (Figure 6b). The cleavage fracture surface is largely characterized as smooth and flat, showing the lack-of-bonding between adjacent layers or tracks.

In addition, some spherical particles were found in the lack-of-bonding area (i.e., smooth and flat area), which were considered as incompletely melted powder particles during the LENS deposition process (Figure 6b). This defect may result from excessive powder injected into the melt pool [15], misalignment of the deposition head [20,38], or inconsistent energy density [32].

**Figure 7:**
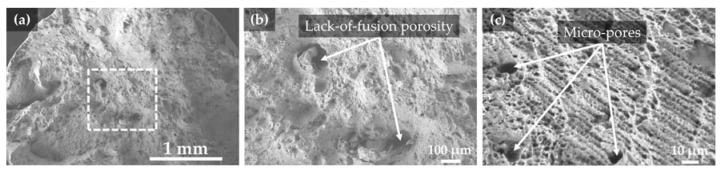
SEM images showing (**a**) overall view of the fracture surface of specimen #4 showing a ductile fracture mode; (**b**) high magnification of a dashed box from (**a**) showing lack-of-fusion porosity; and (**c**) high magnification of the dimple fracture surface showing micro-pores.

Figure 7a shows the overall fracture surface morphology of specimen #4 that has the highest elongation value among the test specimens, which dominantly appeared to be a ductile fracture mode with partial cleavage-type deformation around the outer edge of the fracture surface.

In addition, lack-of-fusion porosity such as a large and irregular shape were found due to localized incomplete melting during the LENS process, which led to the presence of cavities in the as-deposited part (Figure 7b). This may be attributed to insufficient dissipation of the laser energy density into the powder particles since porosity is inversely proportional to the absorbed laser energy per unit length of deposited track [20,24].

A number of micro-pores with small and spherical shapes were also observed in the dimple fracture surface (Figure 7c), which was attributed to the gas entrapped hollow particles in the gas-atomized (GA) powders [17,20,22,38,39]. During the LENS process, the inert gas inside the powders could not escape from the melt pool due to the rapid solidification process, resulting in the formation of micro-pores in the as-deposited IN718 part.

From the fractography analysis above, metallurgical defects found in the fracture surface can be mainly classified into two types: (1) process-induced defects such as incompletely melted particles and lack-of-fusion porosity caused by the instability of the LENS process and (2) material-induced defects such as micro-pores due to hollow GA powder particles. These undesired defects can cause micro-porous coalescence cracks [22] and weaken the mechanical properties by reducing the density and interlayer bonding of the as-built part [40].

While using powders free of gas porosity can eliminate micro-pores in the final specimen, complete melting of the powders with sufficient laser energy is necessary to prevent lack-of-fusion. Even though a lower energy density causes lack-of-fusion porosity, it also leads to a finer microstructure and reduced segregation in the as-deposited IN718 part [24]. Therefore, using optimal processing parameters is of critical importance to have a balance between porosity, refined microstructure, and segregation.

#### 3.3.2. Elemental Segregation and Laves Phase Formation

**Figure 8:**
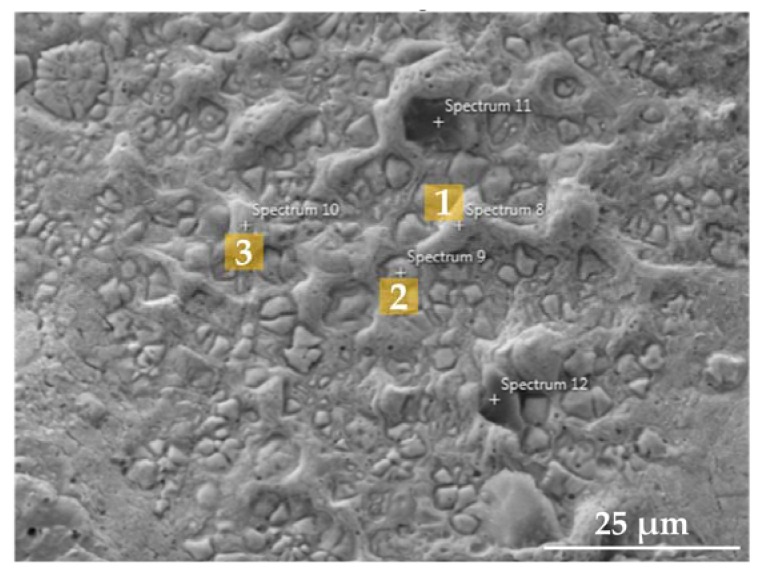
SEM micrograph of the fracture surface for the EDS spot analyses.

From the EDS spot analyses indicated in Figure 8, the quantitative elemental distributions were obtained in Table 2. Notable elemental segregation and Laves phase formation were observed on the cleavage (brittle) fracture area of specimen #2 (Figure 6b).

As seen in Table 2, all the spots analyzed in this area showed a higher weight fraction (wt %) of Nb, Mo, and Ti that are principal elemental constituents of the Laves phase compared to the nominal element composition of the IN718 superalloy. In particular, the Nb concentration in this region ranges from about 19% to 24%, which is much higher than that in the original IN718 composition. In contrast, this region revealed the depletion of Ni, Fe, and Cr that are major constituents of the austenite γ matrix in the IN718 superalloy.

According to the solidification theory [41,42], the primary γ dendrites are formed firstly while segregating Nb and C from the liquid due to their low maximum solubility in the liquid with a dramatic decrease in temperature [43]. As solidification continues, Nb and C become richer until the maximum solubility of Nb and C is reached in the γ matrix, which gives rise to γ and NbC by a eutectic reaction (L→(γ + NbC)) and depletes C with enriched Nb. As the remaining liquid with available Nb continues to form γ dendrites, solidification ends up with a secondary eutectic reaction (L→(γ + Laves)) at last. This eutectic is frequently observed in the IN718 weld part [25,37,44,45,46,47].

Therefore, the presence of Nb-rich Laves on the tensile fracture surface of specimen #2 is correlated with the segregation of highly refractory elements such as Nb, Mo, and Ti during the non-equilibrium solidification process of IN718 [24,45,48]. These solute elements easily tend to segregate from the austenite γ matrix due to the larger atomic radius size requiring more diffusion energy and the greater atomic size mismatch with Ni, Fe, and Cr, which means these constituents have a limited ability to dissolve into the matrix [49,50].

Since the Laves phase is the product of a non-equilibrium eutectic reaction, L→(γ + Laves), at the end of IN718 superalloy solidification, its formation depends on the local non-equilibrium conditions of solidification and the corresponding Nb concentration [42,48].

The Laves phase is a Nb-rich brittle intermetallic compound identified as a (Ni, Fe, Cr)_2_(Nb, Mo, Ti) form [37]. However, it was reported that the stoichiometry of the observed Laves phase is not necessarily matched with that of the expected one, and identical stoichiometry is not required in the Laves phase [51]. The Laves phase is empirically distinguished by highly concentrated Nb wt % ranging from 10% to 30% depending on the different solidification conditions such as heat input and cooling rate [45,52]. Similar elemental composition distribution from the EDS measurement results was also observed from the previous literature [31], indicating that this phase is Laves. Therefore, the observed phase on the fracture surface in this study was confirmed theoretically and empirically as the Laves phase.

This brittle phase can initiate micro-cracks and cause fracture failure upon tensile stress loading, which will significantly weaken the mechanical properties of as-deposited IN718 [50]. As observed in Figure 8 and Table 2, a large amount of Laves phases were distributed in the cleavage and brittle fracture surface area. Furthermore, the tensile ductility and UTS were considerably lower compared to the other specimens, which could be attributed to the presence of the secondary intermetallic Laves phase.

It was reported that the Laves phase is in cohesion with the γ matrix due to the atomic size mismatch, resulting in poor bonding along the interface between the Laves phase and the γ matrix and in turn, crack initiation and propagation along the weakened fracture paths [45,47,53]. Additionally, the Laves phase consumes the principal alloying elements such as Nb, Mo, and Ti which are necessary for precipitation of the strengthening phases such as γ″ (Ni_3_Nb) and γ′ (Ni_3_(Al, Ti)) [35,45]. Therefore, the Laves phase is detrimental to the mechanical properties such as tensile ductility, ultimate tensile strength, fracture toughness, fatigue, and creep rupture [17,35,54,55], which could affect the structural integrity and cause premature failure of critical components during their service [45,56].

The as-deposited part is normally considered to show inferior mechanical performance compared to wrought material due to the negative formation of the Laves phase [31]. To mitigate the segregation and subsequent formation of the Laves phase, the control of process conditions such as fast cooling rate, low heat input, steep thermal gradients, etc., was suggested [45] since segregation is a time and temperature dependent phenomenon and is highly influenced by the cooling rate which could be controlled by the laser power, scanning speed, and scanning path [24]. It is also reported that post processing such as heat treatment could improve the mechanical properties of the as-deposited part by dissolving the detrimental intermetallic phase and precipitating the strengthening phases [24,31,57].

#### 3.3.3. Oxide Formation

**Figure 9:**
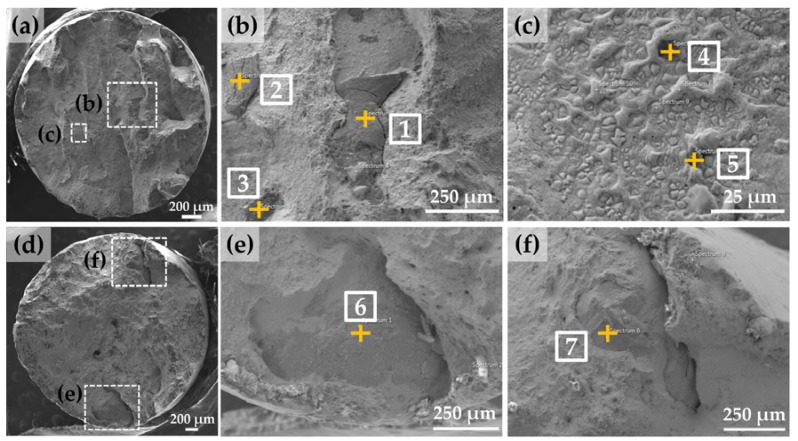
SEM images showing oxide films on the fracture surface. (**a**) overall view of specimen #2; (**b**) high magnification of a dashed box marked as (b) in Figure 9a; (**c**) high magnification of a dashed box marked as (**c**) in Figure 9a; (**d**) overall view of specimen #4; (**e**) high magnification of a dashed box marked as (**e**) in Figure 9d; and (**f**) high magnification of a dashed box marked as (**f**) in Figure 9d.

Figure 9a,d show the morphology of the oxide films on the entire fracture surface of specimens #2 and #4, respectively. Figure 9b,c,e,f reveal high magnification of the corresponding oxides from the dotted areas of specimens #2 and #4, respectively. These were clearly distinguished from the matrix, which appear dark in secondary electron (SE) mode.

As shown in Figure 9b,e,f, there were large and thick oxide films covering the fracture surfaces. It implies that extensive oxidation occurred in the melt pool during the deposition. It was also observed from a previous study [58] that many oxide particles were dispersed in the melt pool, which increased the viscosity in the melt pool under air, and the oxidation reactions in the melt pool continued as the temperature lowered. The Marangoni flows also affect the heat transfer behavior in the melt pool and the oxide shape. When the oxygen level exceeds a certain concentration in the melt pool, surface tension decreases as the temperature decreases due to the increased viscosity. This changes the direction of the Marangoni flows from outward to inward with a relatively deep melt pool shape [59]. Thus a thick oxide layer covered the surface of the liquid melt pool during the LENS process at the higher oxygen content atmosphere [59]. On the other hand, some of the oxides represented as dark phases in Figure 9c were embedded in the Laves/γ eutectic region as mentioned in Section 3.3.2. It was found from the literature [60] that the oxides and intermetallic Laves showed good lattice match and served as nucleation sites for each other.

**Figure 10:**
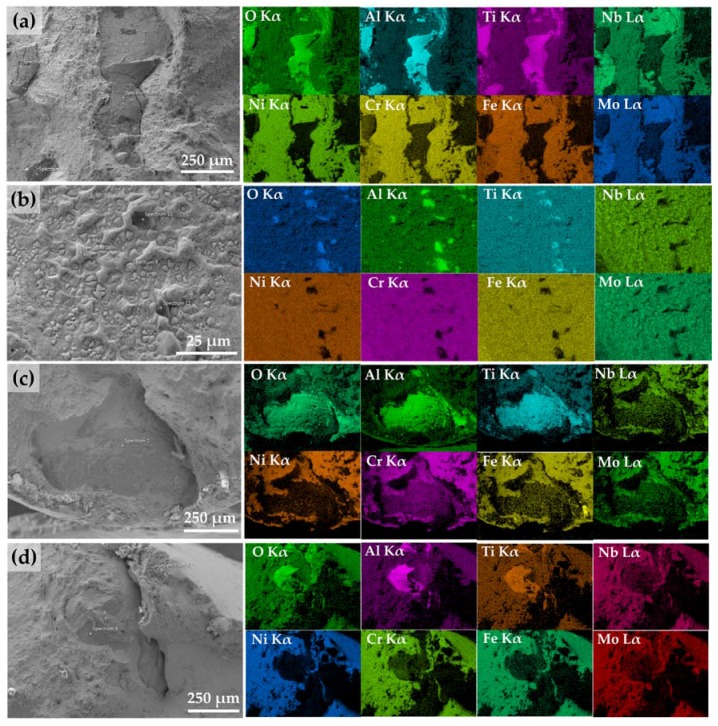
EDS element mapping of the dark phases in the selected areas (**a**) from Figure 9b; (**b**) from Figure 9c; (**c**) from Figure 9e; and (**d**) from Figure 9f, showing enrichment in O, Al, and Ti and depletion of Ni, Cr, Fe, Mo, and Nb.

EDS element mapping in the selected regions from Figure 9b,c,e,f revealed that O, Al, and Ti were highly concentrated, but Ni, Fe, Cr, Nb, and Mo were depleted in the dark phases, as shown in Figure 10a–d.

The quantitative chemical composition on the dark phases of the same areas as marked in Figure 9 was also obtained from the EDS spot analyses in Table 3, which gives further evidence of higher atomic fraction (at %) of O, Al, and Ti contents with a lack of main alloying elements in IN718 such as Ni, Cr, Fe, Nb, and Mo as compared to the γ matrix.

Therefore, both EDS elemental mapping and quantitative spot analyses data simultaneously confirmed that these inclusions were composed primarily of O, Al, and Ti. This implies that aluminum oxide and titanium oxide were formed as a result of the chemical reaction with oxygen molecules in the chamber during the LENS process [61].

It was difficult to obtain the stoichiometry of the oxides from the quantitative composition in Table 3 because of the multiple elements detected. The results of the spot analyses on the oxides were unavoidably affected by the X-ray spectra from the matrix under the oxides due to the increased excitation volume caused by high accelerating voltage [62,63], which made it difficult to determine the ideal chemical formula such as Al_2_O_3_ and TiO_2_. However, these oxide formations can be explained by thermodynamic data as follows.

As shown in Table 3, the major elements of IN718 are Ni, Fe, Cr, Ti, and Al. According to the Ellingham diagram [64,65], these elements strongly tend to become oxidized by forming NiO, FeO, Cr_2_O_3_, TiO_2_, and Al_2_O_3_, respectively. Based on the thermodynamic data of Gibbs free energy, the formation of the oxides at the melting point of IN718 (1336 °C) are in the following order: Al_2_O_3_ > TiO_2_ > Cr_2_O_3_ > FeO > NiO. It explains that the Gibbs free energy of Al_2_O_3_ for oxide formation is the lowest and most stable, and the oxidation of Al forms a continuous Al_2_O_3_ scale quickly, followed by the oxidation of Ti (i.e., TiO_2_). From a thermodynamic perspective, Al_2_O_3_ is the most possible oxide to be formed in IN718, followed by TiO_2_ [60,65,66]. Therefore, it can be reasoned that the observed oxide films would be identified as aluminum oxide (Al_2_O_3_) and titanium oxide (TiO_2_) with evidence of the EDS composition data.

The presence of the oxides in the as-deposited IN718 part can be explained by the difference of thermophysical properties between the oxides and the IN718. The melting point of IN718 is 1336 °C, and the melting points of the oxides such as Al_2_O_3_ and TiO_2_ are 2072 °C and 1843 °C, respectively. Thus, these oxides can hardly be melted during the LENS process. As a result, a number of oxides were formed and trapped into the following layers in the as-deposited IN718 part. In addition, the density of Al_2_O_3_ and TiO_2_ (3.95 g/cm^3^ and 4.13 g/cm^3^) is lighter than IN718 (8.19 g/cm^3^). As a result, the oxides would float on the top of the melt pool in IN718 due to the density difference, which could hinder interlayer bonding [32].

During the LENS process at high temperature, oxygen diffuses into the as-deposited layer along the grain boundary and micro-pores/voids inside the part in which it reacts to form aluminum and titanium oxide, which is called thermally grown oxide [61]. Regardless of the type of oxide, compressive stress is induced in this thermally grown oxide due to the diffusion. As a result, plastic deformation occurs to release the stress within the oxide and along the interface between the oxide and the matrix, which generates intergranular voids due to the grain boundary sliding [67]. These voids and continuous plastic deformations result in the crack initiation and propagation at the stress concentration area [62,67,68].

As observed on the fracture surfaces in Figure 9, the oxide films inhibited metallurgical bonding between layers and across tracks during the LENS process. The interfaces between the oxide films and the matrix showed poor bonding by generating cracks along the interface as well as within the oxide films, which could accelerate the fracture failure of the as-deposited part and in turn adversely affect the mechanical properties.

Since oxides can act as crack initiators, these inclusions in the as-deposited part need to be minimized. The Ellingham diagram can be used to determine the partial pressure of oxygen that is in equilibrium with a metal oxide at a given temperature [65]. If the oxygen partial pressure is higher than the equilibrium value, the metal will be oxidized. According to the Ellingham diagram, the oxygen partial pressure must be lower than 10^−24^ atmospheres for Al and 10^−20^ atmospheres for Ti at the melting point of IN718 (1336 °C) to prevent oxidation. Even high purity shielding argon gas contains impurities such as O, CO, CO_2_, H, and H_2_O, however, the formation of oxide films cannot be avoided completely [69]. Thus further investigation is required to gain an understanding of how to mitigate the detrimental effect of oxidation to ensure successful interlayer bonding during the LENS process of IN718.

### 3.4. Microhardness

**Figure 11:**
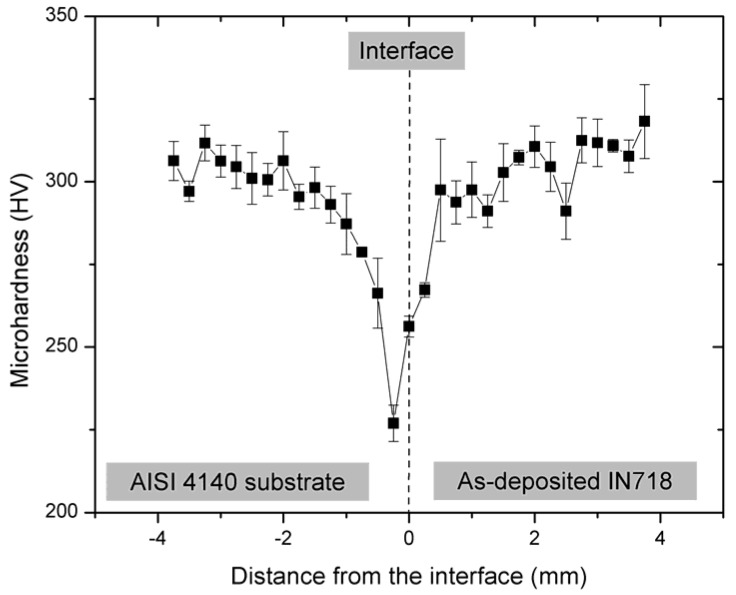
Average microhardness profile of the cross-sectional hybrid specimen including the AISI 4140 substrate (**left**); interface (**middle**); and as-deposited IN718 (**right**).

The average Vickers microhardness profile on the cross-sectional hybrid specimen including the AISI 4140 substrate, interface, and as-deposited IN718 is shown in Figure 11. The average microhardness of the as-deposited IN718 (301.6 ± 13 HV) was higher than that of the substrate (292.0 ± 21 HV). There was a sharp decrease in microhardness near the interface in the substrate, which showed the lowest microhardness (226.9 ± 5 HV) at the HAZ below the interface. This was attributed to microstructural changes and micro-cracks as a result of subsequent heat transfer from the laser-deposited layers due to the repeated heat cycles and thermal stress, as illustrated in Figure 3c.

The overall hardness of the as-deposited IN718 part was not as hard as wrought IN718 (372 HV) and cast IN718 (350 HV) according to AMS 5663 and AMS 5383, respectively. This lower hardness was due to segregation of the principal alloying elements (Nb, Mo, and Ti) and Laves/γ eutectic formation during the rapid solidification of IN718, which depleted these elements necessary for precipitating strengthening phases (γ″ and γ′). Previous studies also reported that the microhardness of as-deposited IN718 was 255 HV [70], 275 HV [71], and 300 HV [72], depending on different processing parameters and conditions. Therefore, proper heat treatment is required to improve the mechanical properties of the as-deposited part by dissolving the detrimental intermetallic Laves phase and precipitating the strengthening phases.

## 4. Conclusions

In this study, IN718 powders were deposited on AISI 4140 substrates using LENS to investigate the compatibility between two dissimilar materials with a focus on interface bonding and tensile fracture behavior of the hybrid specimens. The main findings were obtained as follows.

1. The interface between the AISI 4140 substrate and as-deposited IN718 obtained from the hybrid tensile specimen exhibits good metallurgical bonding.
2. Through the tensile test, all the tensile fractures occurred in the laser-deposited IN718 section of the hybrid specimens, rather than the interface or the substrate. It is implied that the laser-deposited interlayer bond strength is weaker than the interfacial bond strength.
3. From the fractography analysis using SEM and EDS, three major factors affecting the tensile fracture failure of the as-deposited part are (i) metallurgical defects such as incompletely melted powder particles, lack-of-fusion porosity, and micro-pores; (ii) elemental segregation and the Laves phase, and iii) oxide formation.
4. The average microhardness of the as-deposited IN718 was higher than that of the substrate, which is attributed to microstructural changes and micro-cracks in the substrate as a result of the repeated heat cycles and thermal stress from the laser-deposited layers.
5. The fracture failure mechanism is a combination of all these factors, which is detrimental to the mechanical properties and structural integrity, and causes premature failure of critical components during the service.
6. Further investigation is required to gain an understanding of how to mitigate these deleterious effects on the as-deposited IN718 coating/repair part, to ensure successful interlayer bonding during the LENS process.

## Figures and Tables

**Table 1:** Comparison of tensile properties of the test specimens with the substrate and wrought IN718.

Material	UTS (MPa)	YS (MPa)	Elongation (%)
Tensile specimens in this study *	662 ± 49	460 ± 55	14 ± 2
AISI 4140 substrate [34]	720	655	4
IN718 wrought (AMS 5663) [35]	1275	1034	12

* Tensile specimens fractured in the as-deposited IN718 part.

**Table 2:** Elemental composition distribution of the corresponding spots marked in Figure 6 (wt %).

Spot	Al	Ti	Mo	Nb	Cr	Fe	Ni	Total	Phase
1	0.34	1.69	3.49	20.92	14.74	12.94	45.89	100	Laves
2	0.22	1.23	4.92	23.94	13.98	12.86	42.84	100	Laves
3	0.56	1.69	3.04	18.90	14.85	13.16	47.81	100	Laves
Powder	0.30	0.60	2.80	4.70	19.00	19.00	53.60	100	Original

**Table 3:** Elemental composition of the oxides from the EDS analyses at spots 1–7 marked in Figure 9 (at %).

Spot	O	Al	Ti	Nb	Cr	Fe	Ni	Total
1	64.90	24.63	8.44	-	1.12	0.22	0.67	100.00
2	68.39	22.44	6.23	0.12	2.12	0.35	0.35	100.00
3	32.39	25.49	34.25	0.22	4.81	0.98	1.86	100.00
4	56.32	35.89	4.76	-	1.08	0.54	1.41	100.00
5	60.53	34.43	3.62	0.11	0.55	0.22	0.55	100.00
6	61.13	26.14	9.41	0.11	2.77	0.22	0.22	100.00
7	61.98	25.25	8.90	0.14	2.73	0.43	0.57	100.00
